# Literary Notes

**Published:** 1909-09

**Authors:** 


### LITERARY NOTE

The *Trained Nurse* is publishing a series of articles relating to state registration for nurses written by Miss Charlotte A Aikens. It is the aim to obtain unbiased judgment upon this question and the magazine is the medium of information. It is sending out a long series of questions which Miss Aikens desires answered. We regret that lack of space prevents their publication. The August number of the *Trained Nurse* contains the first article which is entitled, A Symposium on Registration. Address, Charlotte A. Aikens, care Lakeside Publishing Co., 114-116 East 28th Street, New York.

